# Theoretical Study of Co-Doping Effects with Different Ions on the Multiferroic Properties of BiFeO_3_ Nanoparticles

**DOI:** 10.3390/ma17174298

**Published:** 2024-08-30

**Authors:** Angel T. Apostolov, Iliana N. Apostolova, Julia M. Wesselinowa

**Affiliations:** 1 Department of Physics, Faculty of Hydrotechnics, University of Architecture, Civil Engineering and Geodesy, Hristo Smirnenski Blvd. 1, 1046 Sofia, Bulgaria; angelapos@abv.bg; 2 Faculty of Forest Industry, University of Forestry, Kl. Ohridsky Blvd. 10, 1756 Sofia, Bulgaria; inaapos@abv.bg; 3 Faculty of Physics, Sofia University “St. Kliment Ohridski”, J. Bouchier Blvd. 5, 1164 Sofia, Bulgaria

**Keywords:** co-doped BiFeO_3_ nanoparticles, multiferroic properties, band gap energy, microscopic model, Green’s function theory

## Abstract

Using a microscopic model and the Green’s function theory, the size and co-doping effects on the multiferroic and optical (band gap) properties of BiFeO_3_ (BFO) nanoparticles are investigated. The magnetization increases, whereas the band gap energy decreases with decreasing nanoparticle size. The substitution with Co/Mn, Nd/Sm, Ce/Ni, and Cd/Ni is discussed and explained on a microscopic level. By the ion co-doping appear different strains due to the difference between the doping and host ionic radii, which leads to changes in the exchange interaction constants for tuning all properties. It is observed that by co-doping with Nd/Sm at the Bi site or with Co/Mn at the Fe site, the multiferroic properties are larger than those by doping with one ion. Moreover, by doping with Ni, the multiferroic properties are reduced. But by adding another ion (for example Ce or Cd), an increase in these properties is obtained. This shows the advantages of the co-doping, its flexibility, and its greater possibility of tuning the multiferroic properties compared to single ion substitution. The band gap energy decreases for all co-dopants. The polarization increases with increasing magnetic field. This is evidence of magnetoelectric coupling, which is enhanced by co-doping with Co/Mn. The observed theoretical results are in good qualitative agreement with the existing experimental data.

## 1. Introduction

Multiferroics are a distinctive class of functional materials that exhibit two or more ferroic orders, such as ferroelectricity, ferromagnetism, and ferroelasticity, within the same phase [1,2]. Additionally, the coupling between magnetism and ferroelectricity in these materials enables the induction of electrical polarization by a magnetic field and vice versa. In recent years, BiFeO_3_ (BFO) has garnered significant attention as a multiferroic material due to its simultaneous ferroelectric and antiferromagnetic properties at room temperature [3]. Its high Curie temperature ($T_{C}$ = 1103 K) and Neel temperature ($T_{N}$ = 643 K) make it an ideal candidate for multifunctional devices [4]. The electronic polarization in BFO is attributed to the free electron pair in the s−p hybrid orbital of Bi^3+^ ions, while the magnetization is due to unpaired electrons in the *d* orbital of Fe^3+^ ions. BFO exhibits a G-type antiferromagnetic order, which is modulated into a spiral spin structure. There exist some significant problems in this compound associated with a high leakage current, very low polarization, crystalline phase stability, high coercive field, and very weak magnetoelectric coupling at room temperature, which limit its practical application in electronic devices [5]. To address these issues, ion substitution with rare earth ions at the Bi sites [6,7] or with transition metal ions at the Fe sites [8,9] has been implemented, impacting the phase structure and multiferroic properties of BFO. Doping BFO nanoparticles (NPs) with various ions can further enhance their multiferroic properties [10]. Additionally, multiferroic nanomaterials have attracted a significant interest due to their applications in multifunctional, low-power consumption, and environmentally friendly devices.

In recent years, to obtain a better magnetoelectric effect in BFO bulk and NPs, many papers have been published, which consider the co-doping effect on their multiferroic properties. Akhtar et al. [11] discussed the efficient magnetoelectric dispersion in Ni and Co co-doped BFO multiferroics and showed that the magnetoelectric effect and the ac conductivity increase. Structural, magnetic, and photocatalytic properties of pure, Ce/Ni, and Cd/Ni co-doped BFO NPs were studied by Kebede et al. [12,13]. The substitution of Mn ions into Fe sites and La into Bi sites of BFO nanofibers resulted in a significant increase in saturation magnetization at room temperature, attributed to the enhanced double exchange interactions of Fe^3+^-O-Fe^2+^ [14]. Additionally, Akhtar et al. [15] studied Pr/Co co-doped BFO NPs for absorber applications. The tuned magnetic, ferroelectric, dielectric, and optical properties of BFO bulk and nanostructures were investigated by co-doping with Ca/Pb [16], Mn/Co [17,18], Nd/Co [19], Y/Mn [20], Ni/Ti [21], La/Co [22], Sm/Co [23], Gd/Co [24], Gd/Zn [25], and Er/Mn [26].

Unfortunately, there are not so many theoretical works that have studied the properties of co-doped BFO materials. Based on first-principles density functional theory calculations, the electronic structures, electric, magnetic, dielectric, and optical properties of Cu/Zn co-doped, X (X = F, N, S) and Cr co-doped, as well as La/Co co-doped BFO were investigated by Rong et al. [27], Zhou et al. [28] and Tariq et al. [29], respectively.

The aim of the present study was to investigate the co-doping ion effects by substitution at an Fe or a Bi site, as well as at both Bi and Fe sites on the multiferroic and optical properties of BFO NPs using a microscopic model and Green’s function theory and to compare the observed theoretical results with the existing experimental data.

## 2. The Model

The multiferroic properties of BFO can be described by using the following Hamiltonian:

(1) $$H=H_{m}+H_{e}+H_{me}.$$

The magnetic properties of ion-doped BFO are characterized by a modified Heisenberg model:

(2) $$\begin{array}{ccc} H_{m} & = & -\sum_{i,j}\left(1-x\right)J_{ij}^{Fe-Fe}S_{i}^{Fe}\cdot S_{j}^{Fe}-\sum_{i,j}x\left(x^{\prime }\right)J_{ij}^{Fe-DI}S_{i}^{Fe}\cdot S_{j}^{DI}\\ & - & \sum_{i}D_{i}{\left(S_{i}^{zFe}\right)}^{2}-g\mu_{B}h\cdot \sum_{i}S_{i}^{Fe}. \end{array}$$

In this context, $S_{i}^{Fe,DI}$ denotes the Heisenberg spin operator for the Fe ion or doping ion (DI) at site *i*. The exchange interaction constants $J_{ij}^{Fe-Fe}$ (which account for both nearest-neighbor and next-nearest-neighbor interactions) and $J_{ij}^{Fe-DI}$ describe the interactions between Fe-Fe and Fe-DI ions, respectively. The parameters *x* and $x^{\prime }$ represent the concentrations of the substituted ions at the Fe and Bi sites, respectively. $D_{i}$ is the single-ion anisotropy constant, and *h* signifies an external magnetic field.

The Ising model in a transverse field (TIM) can describe the ferroelectric properties of BFO driven by displacements of the Bi^3+^ ions from the center of the FeO_6_ octahedra [30]:

(3) $$H_{e}=-\Omega \sum_{i}B_{i}^{x}-\frac{1}{2}\sum_{ij}\left(1-x^{\prime }\right)J_{ij}^{\prime }B_{i}^{z}B_{j}^{z}.$$

Here, $B_{i}^{x}$ and $B_{i}^{z}$ represent pseudo-spin operators, and $J_{ij}^{\prime }$ denotes the nearest-neighbor exchange pseudo-spin interaction. The dynamics of the ferroelectric part is realized by the transverse term with the flipping rate Ω and the operator $B^{x}$. A new coordinate system is obtained by rotating the original one used in (3) by an angle on the xz plane chosen to ensure that $\langle B^{x^{\prime }}\rangle =0$ in the new coordinate system. Blinc and de Gennes [31] proposed the TIM for the description of order–disorder KH_2_PO_4_ (KDP)-type ferroelectrics. Further, the TIM is applied to displacive-type ferroelectrics such as BaTiO_3_ (BTO) [32], too.

The magnetoelectric term that couples the two-order parameters is given by

(4) $$H_{me}=-g\sum_{ijkl}B_{i}^{z}B_{j}^{z}S_{k}\cdot S_{l}.$$

A quadratic magnetoelectric coupling *g* in BFO is assumed due to the large difference between $T_{C}$ and $T_{N}$, $T_{C}>>T_{N}$.

The spontaneous magnetization $M=\langle S^{z}\rangle$ is computed using the method of Tserkovnikov [33] as follows:

(5) $$M=\langle S^{z}\rangle =\frac{1}{N^{2}}\sum_{ij}\left[\left(S+0.5\right)\coth\left[\left(S+0.5\right)\beta E_{mij}\right)]-0.5\coth\left(0.5\beta E_{mij}\right)\right],$$

where *S* represents the spin value, $\beta =1/k_{B}T$. $E_{mij}$ denotes the spin excitation derived from the spin Green’s function $G_{ij}=\ll S_{i}^{+};S_{j}^{-}\gg$ neglecting the transverse correlation functions $\langle S_{i}^{-}S_{j}^{+}\rangle$ and decoupling the longitudinal correlation functions $\langle S_{i}^{z}S_{j}^{z}\rangle \to \langle S_{i}^{z}\rangle \langle S_{j}^{z}\rangle$:

(6) $$\begin{array}{ccc} E_{mij} & = & \frac{\langle \left[\left[S_{i}^{+},H\right],S_{j}^{-}\right]\rangle }{\langle \left[S_{i}^{+},S_{j}^{-}\right]\rangle }=\frac{2}{N}\sum_{m}\left(1-x\right)J_{im}^{Fe-Fe}\langle S_{m}^{zFe}\rangle \delta_{ij}\\ & - & \frac{2}{N}\left(1-x\right)J_{ij}^{Fe-Fe}\langle S_{i}^{zFe}\rangle +2D_{i}\langle S_{i}^{zFe}\rangle \delta_{ij}\\ & + & \frac{2}{N}\sum_{m}x\left(x^{\prime }\right)J_{im}^{Fe-DI}\langle S_{m}^{zDI}\rangle \delta_{ij}-\frac{2}{N}x\left(x^{\prime }\right)J_{ij}^{Fe-DI}\langle S_{i}^{zDI}\rangle +2g\mu_{B}h. \end{array}$$

The magnetoelectric coupling *g* renormalizes the spin exchange interaction constant between the next nearest neighbors

(7) $$J_{eff}^{Fe-Fe}=J^{Fe-Fe}+2gP^{2}cos^{2}\theta .$$

From the Green’s function

(8) $$g_{ij}=\ll B_{i}^{+};B_{j}^{-}\gg$$

is evaluated with the spontaneous polarization *P* as

(9) $$P=\frac{1}{2N^{2}}\sum_{ij}\tanh\frac{E_{fij}}{2k_{B}T}.$$

$E_{fij}$ is the pseudo-spin wave energy:

(10) $$E_{fij}=2\Omega \sin\theta +\frac{1}{2}P_{ij}\left(1-x^{\prime }\right)J_{ij}^{\prime }\cos\theta_{i}\cos\theta_{j}.$$

The pseudo-spin exchange interaction constant $J^{\prime }$ is also renormalized due to the magnetoelectric coupling *g* to $J_{eff}^{\prime }$:

(11) $$J_{eff}^{\prime }=J^{\prime }+2g\left(\langle S^{-}S^{+}\rangle +\langle S^{z}S^{z}\rangle \right)/\cos\theta .$$

In order to evaluate the band gap energy, the s−d(f) model is needed. This model was proposed for magnetic semiconductors by Nagaev [34]. Therefore, to the Heisenberg Hamiltonian (2) was added the following two terms: $H_{el}$ and $H_{m-el}$. $H_{el}$ is the Hamiltonian of the conduction band electrons:

(12) $$H_{el}=\sum_{ij\sigma }t_{ij}c_{i\sigma }^{+}c_{j\sigma }+\frac{1}{2}\sum_{ijkl,\sigma \sigma^{\prime }}v\left(ijkl\right)c_{i\sigma }^{+}c_{j\sigma^{\prime }}^{+}c_{k\sigma^{\prime }}c_{l\sigma }.$$

$t_{ij}$ is the hopping integral, *v* is the Coulomb interaction, and $c_{i\sigma }^{+}$ and $c_{i\sigma }$ are Fermi-creation and -annihilation operators.

The s−d coupling term $H_{m-el}$ reads

(13) $$H_{m-el}=\sum_{i}I_{i}S_{i}s_{i}.$$

*I* represents the s−d(f) interaction constant, while $s_{i}$ are the spin operators of the conduction electrons at site *i*, which can be defined as $s_{i}^{+}=c_{i+}^{+}c_{i-}$, $s_{i}^{z}=\left(c_{i+}^{+}c_{i+}-c_{i-}^{+}c_{i-}\right)/2$.

The band gap energy is determined by the difference between the valence and conduction bands:

(14) $$E_{g}=\omega^{+}\left(k=0\right)-\omega^{-}\left(k=k_{\sigma }\right).$$

The electronic energies

(15) $$\omega^{\pm }\left(k\right)=\epsilon_{k}-\frac{\sigma }{2}I\langle S^{z}\rangle +\sum_{k^{\prime }}\left[v\left(o\right)-v\left(k-k^{\prime }\right)\right]\langle n_{k^{\prime }-\sigma }\rangle$$

are calculated from Green’s function $g\left(k\sigma \right)=\ll c_{k\sigma };c_{k\sigma }^{+}\gg$, σ=±1. The occupation number distribution is given by $\langle n_{k^{\prime }\sigma }\rangle$. $\langle S^{z}\rangle$ represents the magnetization.

All quantities need to be calculated self-consistently.

## 3. Numerical Results and Discussion

The numerical calculations were performed with a computer program in Java. A self-consistent iterations method was used. As inputs data for the first iteration, the model parameters given below were used. In any subsequent calculation of the input data, the results of the previous calculation were used. The calculations continue until the difference between two consecutive iterations does not exceed a predetermined small enough value.

For the evaluation of the different properties of BFO, we used the following model parameters: $J_{1}^{Fe-Fe}$ = 55 K, $J_{2}^{Fe-Fe}$ = −115 K, *D* = 0.2 K, $J^{\prime }$ = 235 K, Ω = 20 K, *g* = 10 K, *I* = 0.5 K, *v* = 0.3 eV, *S* = 2.5 for the magnetic spins, and *S* = 0.5 for the pseudo-spins.

### 3.1. Size Dependence of Magnetization and Band Gap Energy in BFO

**Figure 1:**
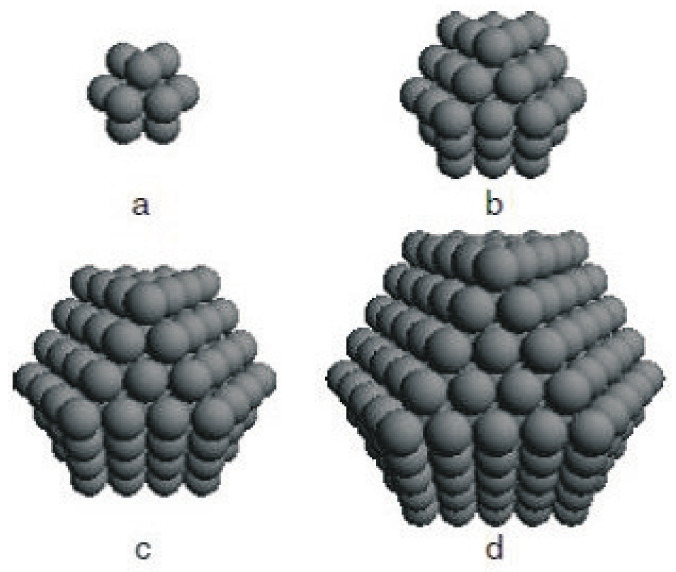
Array of multiferroic NPs composed of different shells. Each sphere represents a spin situated in the center, where (**a**) consists of one central spin plus N = 1 shell, (**b**) N = 2, (**c**) N = 3, and (**d**) N = 4.

An NP is defined by fixing the origin at a certain Fe-spin at the center of the particle and including all other spins within the particle in shells (see Figure 1). The shells are numbered as n=1,...,N, where n=1 denotes the central spin and n=N represents the surface shell of the system. The NP has an icosahedral symmetry, i.e., in the first shell, there are 12 spheres; in the second there are 42 spheres; and in the third shell, there are 92 spheres; and so on. The surface effects are included by different exchange interaction parameters $J_{s}$ within the surface layer (n=N) compared to the bulk one *J*. Typically, BFO NPs are spherical with sizes of 10–50 nm and distance between the shells of about 0.2 nm.

**Figure 2:**
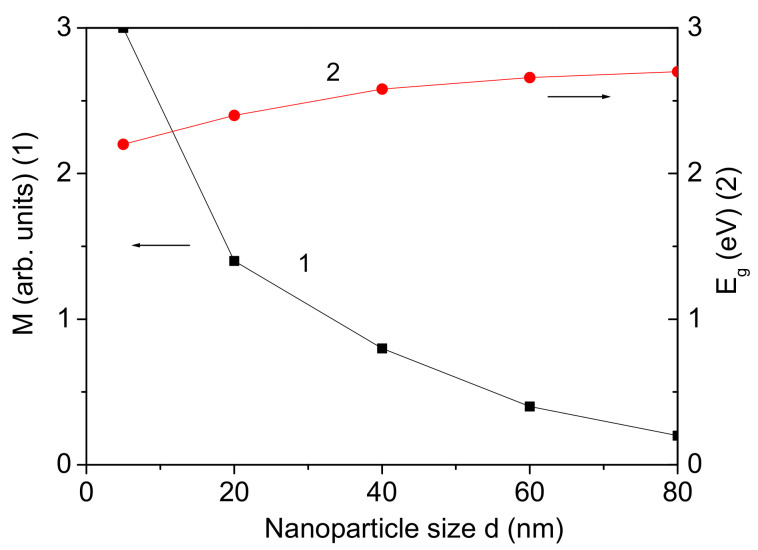
Size dependence of the magnetization *M* (1) and the band gap energy $E_{g}$ (2) for pure BFO, $J_{s}=1.2J$.

BFO is well known as a multifunctional material having excellent ferroelectric, ferromagnetic, piezoelectric, and semiconductor properties [35]. BFO is a type-I multiferroic material that simultaneously exhibits ferroelectricity and antiferromagnetism with high ordering temperatures: an antiferromagnetic Neel temperature $T_{N}$ = 640 K and a ferromagnetic Curie temperature $T_{C}$ = 1100 K. Although the magnetoelectric coupling in bulk BFO is limited due to low polarization and a high leakage current, it is significantly enhanced in thin films [36]. Initially, the size-dependent behavior of the magnetization *M* in a BFO NP is investigated. The BFO demonstrates a G-type antiferromagnetic order, which transitions to a spiral spin structure and undergoes a phase transition near $T_{N}$ = 640 K [4]. Antiferromagnetic nanostructures exhibit significantly enhanced magnetization *M* and coercive fields $H_{c}$ below $T_{N}$ compared to their bulk counterparts. This enhancement is attributed to uncompensated surface spins, which cause a reduction in the lattice parameters of the BFO NPs compared to those of the bulk BFO, to a compressive strain, and to a larger exchange interaction constant on the surface $J_{s}$, that is, to a larger exchange interaction constant of the NP $J_{NP}$ compared to that of the bulk compound *J*, $J_{s}>J$, $J_{NP}>J$. The exchange interaction constant $J_{ij}\equiv J\left(r_{i}-r_{j}\right)$ is inversely proportional to the distance between spins and the lattice parameters. It can be seen from Figure 2, curve 1, that *M* increases with decreasing *d* (for $J_{s}>J$), i.e., there appears to be weak ferromagnetism, which is in agreement with the experimental data of Carranza-Celis et al. [37] and Cardona-Rodriguez for BFO NPs [38]. This result means that the magnetoelectric coupling increases, enabling the tuning of ferroic properties in BFO through NP size.

Unfortunately, the data of the band gap $E_{g}$ in BFO are inconsistent. The band gap value $E_{g}$ of bulk BFO is reported to vary between 2.7 and 3.2 eV [35] or between 2.08 and 2.7 eV [39]. Lima et al. [40], using both collinear and non-collinear spin density functional theory (DFT), calculated a band gap value of 2.6 eV. The size dependence of the band gap energy $E_{g}$ is also presented in Figure 2, curve 2. $E_{g}$ decreases with decreasing NP size *d*, which is attributed to the competing effects of microstrain, oxygen defects on the surface, and Coulomb interactions of BFO NPs. This can also be seen from Equations (14) and (15). As the magnetization *M* increases, the band gap energy $E_{g}$ decreases with decreasing NP size *d*. This trend is consistent with experimental observations by Mocherla et al. [41], who reported a decrease in the band gap $E_{g}$ from 2.32 eV to 2.09 eV in BFO NPs, and Sharma et al. [42], who noted a reduction in the band gap $E_{g}$ from 2.5 eV to 2.0 eV with decreasing NP size. A similar behavior was observed experimentally by Mocherla et al. [41] in BFO NPs. The band gap energy $E_{g}$ decreased from 2.32 eV to 2.09 eV. Sharma et al. [42] reported a tuning of the band gap $E_{g}$ with decreasing NP size from 2.5 eV to 2.0 eV. Additionally, Sando et al. [43] found that the band gap energy $E_{g}$ of BFO thin films is also smaller than that of bulk BFO.

### 3.2. Co-Doping Substitution Effect on the Magnetization and Polarization in BFO NPs

Doping BFO with ions that have different ionic radii compared to the host ions induces varying strains, which alter the exchange interaction constants. These constants are inversely proportional to the distance between the spins and to the lattice parameters. In the doped states, the exchange interaction constants, denoted as $J_{d}$ and $J_{d}^{\prime }$, can be larger or smaller compared to those in the undoped material, denoted as *J*, $J^{\prime }$, depending on whether the strain is compressive or tensile. These changes enable the modulation of all properties of BFO.

The calculations within the framework of the present model are made below the solid-state solubility limit because above this limit, clusters of the doping ions and a metastable supersaturated solution are formed.

### 3.3. Co-Doping at the Bi Site

**Figure 3:**
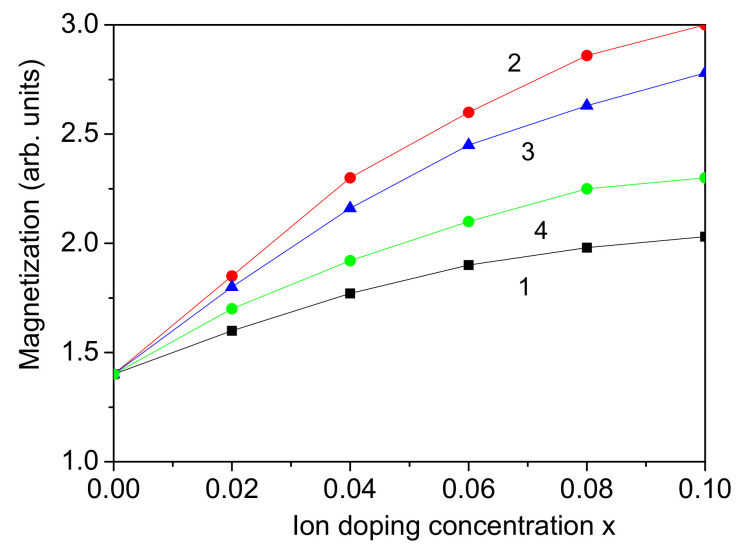
Co-doping concentration dependence of the magnetization *M* for a BFO NP, *d* = 20 nm, for different ion combinations: (1) Nd-Sm (at Bi site); (2) Co-Mn (at Fe site); (3) Cd-Ni (at Bi and Fe sites); (4) Ce-Ni (at Bi and Fe sites).

**Figure 4:**
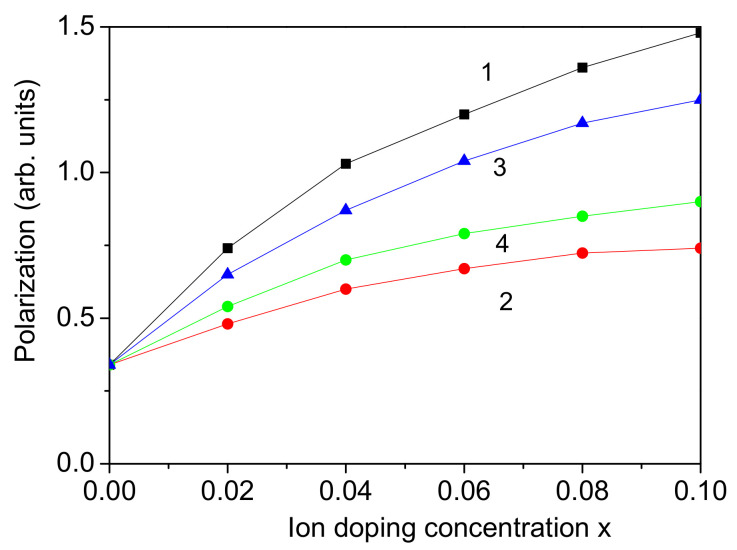
Co-doping concentration dependence of the polarization *P* for a BFO NP, *d* = 20 nm, for different ion combinations: (1) Nd-Sm (at Bi site); (2) Co-Mn (at Fe site); (3) Cd-Ni (at Bi and Fe sites); (4) Ce-Ni (at Bi and Fe sites).

Firstly, we considered the case of co-doping at the Bi site, for example, with the rare earth ions Nd^3+^ and Sm^3+^, which were substituted in equal amounts [44]. Their ionic radii were 1.123 $\dot{A}$ and 1.098 $\dot{A}$, respectively, which are smaller than the ionic radius of Bi^3+^−1.17 $\dot{A}$. This substitution causes a compressive strain leading to the relation $J_{d}>J$, $J_{d}^{\prime }>J$. Enhanced magnetization *M* and polarization *P* are observed in a BFO NP. The results are presented in Figure 3, curve 1, and Figure 4, curve 1. A similar increase in the magnetic and ferroelectric properties in Nd/Sm co-doped bulk BFO was reported by Wang et al. [44]. Bismibanu et al. [45] also demonstrated that Pr and Dy dopant enhance the ferroelectric and magnetic properties of BFO at low concentrations of Dy, suggesting that these materials are promising for multiferroic applications. By doping with one dopant, *P* and *M* increase, too, but by adding the second one, the effect is larger; by co-doping, the increase is larger. Moreover, the co-doping of rare earth dopant (for example with Nd and Nb) could also be made at the A and B sites of BFO as shown by Wang et al. [46], the case of which is not considered here. When one of these rare earth dopants has a larger ionic radius than the Bi ion, i.e., it causes a tensile strain and reduces the multiferroic properties, then the doping concentration of the two co-doped ions plays an important role. But because this is the first theoretical investigation using a microscopic model and Green’s function theory, this case is not considered here.

### 3.4. Co-Doping at the Fe Site

As the next two transition metal ions are substituted at the Fe site, for example, Co^3+^ and Mn^3+^, which are substituted in equal amounts [17,18], the radii of the dopant in the high-spin state are 0.61 $\dot{A}$ and 0.65 $\dot{A}$, respectively. The ionic radius of the host Fe^3+^ ion is 0.645 $\dot{A}$, which is very close to that of Mn^3+^. But as expected, the lattice parameters of the co-doped compound decrease, and the volume decreases, as reported by Marzouli et al. [18]. The difference between the Co and Fe ions is much greater than that between Mn and Fe, so that the influence of the Co ions is much greater than that of the Mn ions and causes a compressive strain. This leads to an increase in the exchange interaction constants $J_{d}$ and $J_{d}^{\prime }$ at the doped states in the BFO NP. Therefore, enhanced magnetization *M* and polarization *P* are observed, i.e., an enhanced magnetoelectric effect. The results are presented in Figure 3 and Figure 4 (curves 2), which are in agreement with the experimental data of Marzouki et al. [18] and Dhanalakshmi et al. [17] for Co and Mn co-doped BFO—bulk and NPs, respectively. Within this model can also be considered Ni/Co co-doped BFO NPs or other combinations of transition metal ions, and we would again obtain enhanced magnetization *M* and polarization *P*, in alignment with Akhtar et al. [11] who studied Ni/Co co-doped BFO.

It is observed that the increase in the magnetization *M* is stronger by co-doping with transition metal ions than with rare earth ones (see Figure 3 and Figure 4, curves 1 and 2), whereas by the polarization *P*, it is vice versa.

In the Co/Mn co-doped BFO NP, we also obtain an increase in the ferroelectric and magnetic phase transition temperatures, $T_{C}$ and $T_{N}$ (not shown here). This is due to the Co ions. $T_{N}$ increases more than $T_{C}$. The origin is that the exchange interaction *J* depends on the Fe-O-Fe bond angle and the Fe-Fe distance. Consequently, substituting Fe with Co or Mn should change the magnetic exchange interaction constant *J* more than the exchange pseudo-spin interaction one $J^{\prime }$. The second one is also changed through the magnetoelectric coupling *g*. Moreover, the Neel temperature $T_{N}$ increases additively by adding more Co ions in the Co/Mn co-doped BFO.

### 3.5. Co-Doping at Both Bi and Fe Sites

Next, we consider the case where the doping ions substitute both the A and B sites, for example, with Ce or Cd ions at the Bi site and Ni ions at the Fe site, Bi_1−*x*_Ce(Cd)_*x*_Fe_1−*x*_Ni_*x*_O_3_ [12]. The ionic radii of the Cd^2+^ (0.87 $\dot{A}$) and Ce^3+^ (1.14 $\dot{A}$) ions are smaller in comparison with the ionic radius of the Bi^3+^ ion (1.17 $\dot{A}$), whereas the ionic radius of the Ni^2+^ ion (0.690 $\dot{A}$) is larger than that of the host Fe^3+^ ion (0.645 $\dot{A}$). The lattice parameters of the co-doped BFO NP decrease as a result of the smaller ionic radii of the dopant, leading to compressive strain in the NP. This is confirmed by the experimental study of Kebede et al. [12] in Ce/Ni and Cd/Ni co-doped BFO NPs. In the present model, we must choose the relations $J_{d}>J$ and $J_{d}^{\prime }>J^{\prime }$. Thus, we obtain an increase in the magnetization *M* and the polarization *P*, i.e., an enhanced magnetoelectric effect. The results are demonstrated in Figure 3 and Figure 4 with curves 3 and 4. It can be seen that the behaviors of *M* and *P* are different by doping with Ce or Cd. This is due to the larger difference between the ionic radii of Cd and Bi ions than those of Ce and Bi.

The Ce(Cd)/Ni co-doped BFO is an interesting case. When BFO NPs are doped solely with Ni ions, tensile strain occurs due to the larger ionic radius of Ni compared to that of Fe. This results in a slight reduction in both magnetization *M* and polarization *P*. But regarding the co-doping effect, doping with Ni and Ce or Cd together causes a compressive strain and changes the behavior of *M* and *P*, as they increase. This is one of the important meanings of the co-doped substitution in the materials.

**Figure 5:**
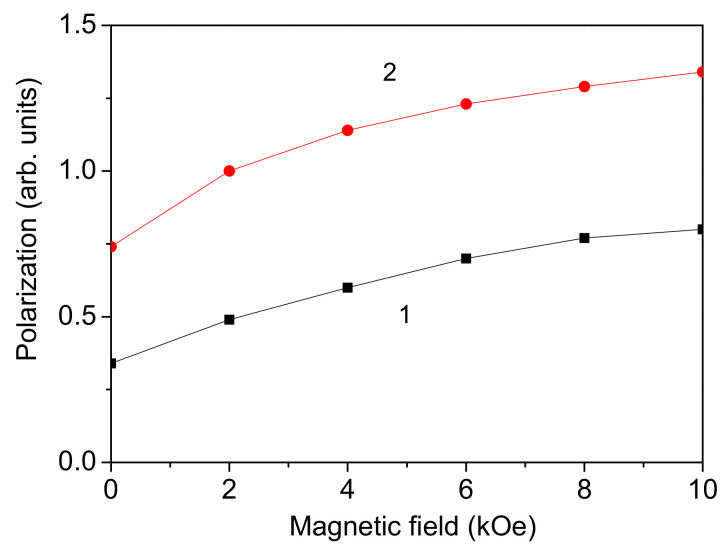
(Color online) Magnetic field dependence of the polarization *P* in a pure BFO NP (1) and a Co-Mn (at Fe site) co-doped BFO NP (2), *d* = 20 nm, *x* = 0.1.

It is observed that the polarization *P* increases with increasing external magnetic field *h* for pure and co-doped BFO NPs (see Figure 5 for a pure and Co/Mn co-doped BFO NP), which shows their multiferroic properties. The obtained increase in the polarization *P* with *h* for all co-doping combinations confirms the enhanced magnetoelectric coupling and magnetoelectric effect in these co-doped BFO NPs. A strong enhancement in the magnetoelectric coupling for Yb and X co-doped BFO (X = Nb, Mn, Mo) in comparison with the undoped BFO was demonstrated by Lakshmi et al. [47].

### 3.6. Co-Doping Substitution Effect on the Band Gap Energy in BFO NPs

**Figure 6:**
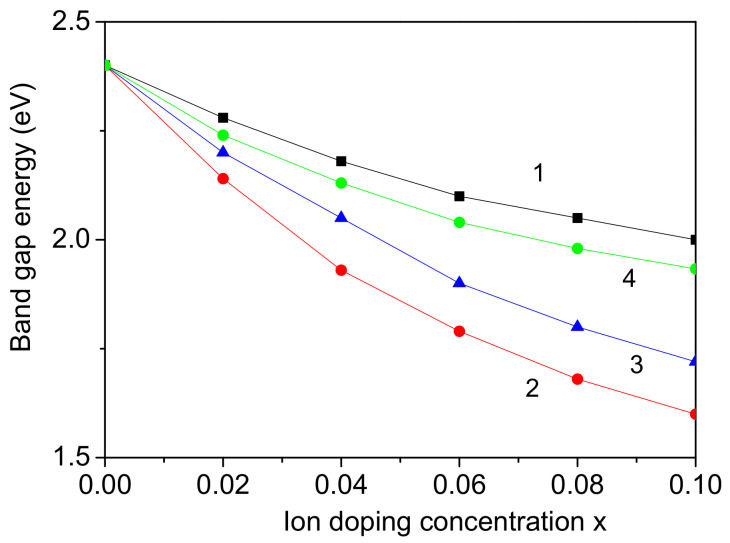
Co-doping concentration dependence of the band gap energy $E_{g}$ for a BFO NP, *d* = 20 nm, for different ion combinations: (1) Nd-Sm (at Bi site); (2) Co-Mn (at Fe site); (3) Cd-Ni (at Bi and Fe sites); (4) Ce-Ni (at Bi and Fe sites).

From Equations (14) and (15), the band gap energy $E_{g}$ in pure and co-doped BFO NPs is calculated. It can be seen that there is a connection between the magnetization *M* and the band gap energy $E_{g}$, as can be expected in magnetic semiconductors. When the magnetization *M* increases, the band gap energy $E_{g}$ decreases. The results for the observed $E_{g}$ for different co-doping cases are shown in Figure 6. The band gap $E_{g}$ decreases for all co-doping cases. This behavior was confirmed for the case of Cd/Ni and Ce/Ni co-doping in BFO NPs at the Bi and Fe sites by Kebede et al. [12]. They reported a tuning of the band gap by Cd/Ni co-doping from 2.10 to 1.75 eV and by Ce/Ni from 2.10 to 2.03 eV. A larger reduction of the band gap energy is also observed for the case of Cd/Ni co-doping in comparison with the case of the Ce/Ni co-doped BFO NP. This is an indirect evidence, that the proposed model and applied method work. Ramachandran and Rao [48] reported a reduced value of $E_{g}$ in Ba/Ca co-doped BFO at the Bi site. A decrease in $E_{g}$ was obtained by co-doping at both Bi and Fe sites by many authors, for example, with Sm/Mn from 2.08 to 1.45 eV [49], with Eu/Mn from 2.40 to 1.49 eV [50], with Gd/Co from 2.23 to 1.77 eV [51], with Nd/Nb from 2.16 to 2.05 eV [46], with Er/Cr from 2.49 to 1.94 eV [52], etc. Using engineering defects to adjust the electronic structure and band gap energy of a semiconductor is a powerful method for tailoring its band structure and charge carrier dynamics, leading to improved photocatalytic performance. These co-doped BFO NPs can be used for photovoltaic devices.

## 4. Conclusions

In conclusion, the magnetization *M*, polarization *P*, and band gap energy $E_{g}$ of pure and co-doped BFO NPs were investigated using a microscopic model and Green’s function theory. The magnetization increased, whereas the band gap energy decreased with decreasing NP size. An increase in *M* and *P* and a decrease in $E_{g}$ with increasing co-dopant concentration (such as Co/Mn, Nd/Sm, Ce/Ni, Cd/Ni) were observed. It was shown that by the substitution of two rare earth elements at the Bi site (for example Nd/Sm) or two transition metal ions at the Fe site (for example Co/Mn), the magnetoelectric properties would be greater than those of the doped BFO NPs with only one ion or of pure BFO NPs. In addition, if one doping ion reduced the magnetoelectric properties, such as Ni, then by adding another rare earth ion with a smaller radius than that of Bi (for example Ce or Cd), i.e., causing a compressive strain, a final effect of an increase in the magnetization *M* and polarization *P* was obtained. The band gap energy $E_{g}$ decreased for all co-dopant.

The magnetoelectric effect in all samples was noticed by plotting the polarization with respect to the magnetic field. It was observed that the polarization increased with increasing external magnetic field, which is evidence of magnetoelectric coupling. This coupling was enhanced by co-doping of Co-Mn ions. This was the aim of this investigation: to discuss the possibilities to enhancing the magnetoelectric effect.

It should be noted that here, we only performed a qualitative comparison with the experimental data. Moreover, there are a lot of combinations of doping ions, which have different radii and can be in different doping concentrations and, thus, can tune the multiferroic properties of BFO NPs. Substitution with two or more ions allows for a more effective and flexible modification of the different properties of BFO NPs required for their multiple applications.

In recent years, studies on ternary and quaternary solid solutions of BFO have been published [53,54,55]. This problem could be studied in a future paper.

## Data Availability

The original contributions presented in the study are included in the article, further inquiries can be directed to the corresponding author.
